# Association between ethical leadership, ethical climate and organizational citizenship behavior from nurses’ perspective: a descriptive correlational study

**DOI:** 10.1186/s12912-020-0408-1

**Published:** 2020-03-04

**Authors:** Soudabeh Aloustani, Foroozan Atashzadeh-Shoorideh, Mansoureh Zagheri-Tafreshi, Maliheh Nasiri, Maasoumeh Barkhordari-Sharifabad, Victoria Skerrett

**Affiliations:** 1 Student Research Committee, School of Nursing and Midwifery, Shahid Beheshti University of Medical Sciences, Tehran, Iran; 2 Department of Psychiatric Nursing, School of Nursing & Midwifery, Shahid Beheshti University of Medical Sciences, Vali-Asr Avenue, Cross of Vali-Asr and Hashemi Rafsanjani Highway, Opposite to Rajaee Heart Hospital, Tehran, 1996835119 Iran; 3 Department of Biostatistics, School of Allied Medical Sciences, Shahid Beheshti University of Medical Sciences, Tehran, Iran; 4 Department of Nursing, School of Medical Science, Yazd Branch, Islamic Azad University, Yazd, Iran; 5 School of Nursing and Midwifery, Birmingham City University, Birmingham, UK

**Keywords:** Ethics, Leadership, Climate, Citizenship behavior, Nurse

## Abstract

**Background:**

Ethical leadership plays an important role in improving the organizational climate and may be have an effect on citizenship behavior. Despite the growing emphasis on ethics in organizations, little attention to has been given this issue. The purpose of this study was to identify ethical leadership, an ethical climate, and their relationship with organizational citizenship behavior from nurses’ perspective.

**Methods:**

In this descriptive correlational study, 250 nurses in twelve teaching hospitals in Tehran were selected by multistage sampling during 2016–2017. The data were collected using Ethical Leadership Questionnaire, Hospital Ethical Climate Survey, and Organizational Citizenship Behavior Scale.

**Results:**

The findings showed a significant correlation between ethical leadership in managers, organizational citizenship behavior (*P* = 0.04, r = 0.09) and an ethical climate (*P* < 0.001, r = 0.65). There was a significant correlation between an ethical climate and nurses’ organizational citizenship behavior (P < 0.001, r = 0.61). The regression analysis showed that ethical leadership and an ethical climate is a predictor of organizational citizenship behavior and confirms the relationship between the variables.

**Conclusion:**

Applying an ethical leadership style and creating the necessary conditions for a proper ethical climate in hospitals lead to increased organizational citizenship behavior by staff. To achieve organizational goals, nurse managers can use these concepts to enhance nurses’ satisfaction and improve their performance.

## Background

Nurses are vital components of health-care systems, forming the largest group of professionals in a hospital [1]. As in many other countries, nurses in Iran are exposed to challenges such as short staffing, heavy workload, undefined responsibilities, shortage of equipment, and low pay [2], poor social status, and the difficulties of negotiating the gap between theory and practice [3], all of which ultimately influence the provision of high quality nursing care [3]. These conditions require a willingness to perform tasks beyond the defined duties and responsibilities, a phenomenon referred to as the “concept of organizational citizenship behavior” in the related literature [4, 5].

Organizational citizenship behavior (OCB) consists of a collection of voluntary behaviors which are not part of the individual’s formal duties. OCB is performed by the personnel without being directly considered by the formal progression system of the organization, yet, it leads to effective and improved fulfillment of organizational roles and responsibilities [4]. These include behaviors that employees voluntarily offer in accordance with their personal choices [6]. OCB is one of the most important factors in determining nurses’ behaviors, attitudes, and interactions to provide high quality services [7]. Altruism, conscientiousness, humility and courtesy, civic virtue, and sportsmanship are signs of the presence of OCB [8]. These voluntary extra-role behaviors may form on the basis of an ethical climate (EC) perceived directly or indirectly by the personnel [4].

EC is the common understanding of various activities and ethical procedures, which also contains an ethical content [9]. The EC is reflected in organizational policies, and is associated with ethical consequences [10]. The EC determines the ethical values and behavior of the organization that influence the ethics of employees. Thus, morally, employees are more likely to be influenced by the organizational climate than their teamwork climate [11, 12]. EC and some variables determine the degree to which decisions are made in the organization on the basis of ethical criteria [13], and provides a framework for ethical decision-making in clinical settings [14]. Consequently, an EC may affect the organizational goals positively via reinforcing the actions that meet or extend the ethical standards leading to better professional performance and improved commitment of the staff [15]. Research has shown that ethical climate has a positive and significant relationship with sportsmanship, civic virtue, and humility [16].

On the other hand, ethical leadership (EL) plays an important role in creating an ethical climate [17]. EL determines the effectiveness of leadership, Employees’ desire for more effort [18], ethical guidance, and occupational satisfaction in the staff through prioritizing moral and ethical codes [19, 20]. Ethical leaders act as role models, establishing clear ethical standards and acting on them. Also, they clarify the ethical standards for their employees and reinforce their behavior in accordance with predetermined ethical standards by rewarding them and counselling those who failed [21]. Employees who have accepted their leader as role model, exhibit behaviors in favor of the organization [21, 22], and encourage them to do more OCB. In other words, the reinforcing nature of ethical leaders make staff do More (frequently) OCB and ethical leaders promote some ethical behaviors by using reward and support to reinforce them, and reinforcement plays an important role in determining modeling effectiveness [23]. Thus, OCB is nurtured despite ethical leadership in the organization [24–26], and ethical leaders can play a significant role in enhancing OCB performance because they have the ability to inspire employees’ perceptions of fairness and integrity [23, 27, 28].

Effective leadership motives nurses to deliver high quality care. An ethical approach is indispensable to leadership in nursing [19, 20]. EL requires an ethical outlook, which is made visible through personal actions and interpersonal relations among the members of the team. It is also encouraged by mutual relations [29], and is defined as an attempt to spread justice, respect for other persons’ individual characteristics, and combining features of honesty and truthfulness [30]. Leaders should not just be concerned with their own benefits; rather, they should be aware of the consequences of decision-making for all individuals [31]. Nursing leaders have considered the ethical behavior as an important organizational issue. The leader ought to play a key role in improving ethical behaviors and climate [32]. When nurses feel they can approach their leaders, having confidence that they will make good decisions and resolve their problems, this will create a climate in which the staff are satisfied with their jobs and feel more commitment to their organization [15].

Social cognitive theory (SCT) suggests that one’s beliefs and motivations are formed on valuable Judgments [33, 34]. SCT is used as a theoretical underpinning to improve the understanding of the relationships between EL, EC and OCB. As leadership styles such as EL is one of antecedents of EC and it can lead to many outcomes i.e. OCB, this framework has been used for this research [35].

Research in this field has demonstrated that EL exerts a considerable effect on EC and personnel’s’ ethical behavior, and EL is recognized as a predictor of EC [11, 12], so, EC is positively correlated to the ethical behavior of the staff [36]. Although scholars agree that leaders play an important role in shaping the moral climate [11, 12, 37, 38], there is not much empirical evidence regarding the relationship between ethical leadership and ethical climate [37].

In this respect, other scholars believe that promotion of EC in health-care systems leads to better responses of nurses to ethical tensions and other causes of dissatisfaction in the work environment [13, 39]. Citizenship behavior is one of the outcomes of leadership, which results in greater organizational productivity [40], and enhances the efficacy of the organization. Additionally, it seems that EL exerts some effects on the positive attitudes of the staff such as organizational commitment, and occupational satisfaction leading to increased rate of OCB of the personnel [41]. Wu et al. assert that although various studies confirm the correlation between EL and variables such as OCB. Despite such rich results, little attention has been paid to the correlation between EL and social responsibility of the organization [42].

The Islamic Republic of Iran is a developing country with a population of about 80 million people located in the Middle East. Islam is the formal religion of this country, and an integration of Iranian-Islamic culture forms the Iranian identity and nature [29]. The Iranian civilization emphasizes the observation of ethical behavior, meritocracy, justice-based rights and fair payments, etc. with the Cyrus Chart of Ethics, dating from the time of the Neo-Babylonian Empire in 539 BC, being seen by many as establishing unprecedented principles of human rights. Islam, which is based on ethical principles of human nature provides us with a plethora of ethical and moral teachings, and emphasizes the observation of individuals’ rights with any position, religion, race, or ethnicity [43]. The entrance of religious disciplines and cultural beliefs of the Iranian nation in the health-care system led to the highlighting of ethical issues in the patient care protocol [29].

The nursing workforce at various levels is estimated to be around 150,000 in Iran, forming a considerable part of the health-care system personnel [29], while we need 500,000 nurses for caring of patients in hospitals [44]. Iranian nurses are almost 78.5% female and 21.5% male. Most of Iranian nurses (72%) have bachelor’s degree, master’s degree, and PhD degree [45]. Many Iranian nurses are not satisfied with their work due to an overwhelming workload, insufficient time and inadequate resources [2], inappropriate work conditions, lack of support, and discrimination in payments [46]. Given the issues mentioned above, this study embarked on investigating the correlation between nursing managers’ EL from the nurses’ perspective with EC and nurses’ OCB.

### Aim

**Fig. 1:**
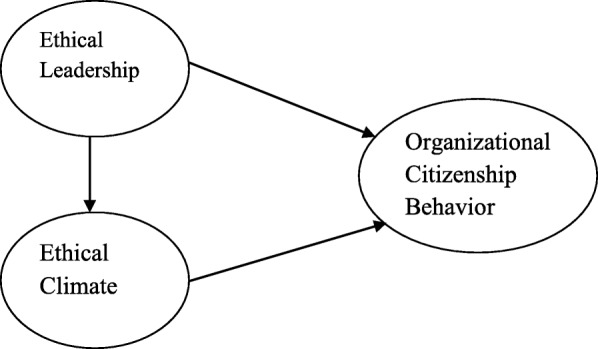
Research conceptual model

This study investigated the correlation between nursing managers’ EL from nurses’ perspective and EC and OCB. For this aim of study, a structural model of this study (Fig. 1) presented, and a hypothesis has been examined to test accuracy of this model.

Part 1: EL of nursing managers is related to the OCB from the nurses’ viewpoint.

Part 2: EC is related to OCB from the nurses’ viewpoint.

Part 3: EL of nursing managers is related to the EC from the nurses’ viewpoint.

## Methods

### Research design

This descriptive correlational study was conducted in twelve teaching hospitals of Shahid Beheshti University of Medical Sciences (SBMU) during 2016–2017. Sampling was done by multistage sampling. That way, first, all teaching hospitals in Tehran affiliated SBMU were classified into five groups based on geographical region and the sample size was distributed in proportion to the size of each group. Then, five hospitals were randomly selected from each group and the number of participants was selected from each group in proportion to the hospital size. A total of 250 nurses working at the teaching hospitals of SBMU, who were selected randomly, participated in this study (response rate = 87%). The participants were asked to answer voluntarily and anonymously. Inclusion criteria of participants in this study were a minimum one-year experience in nursing, holding a BSc. or MSc degree in nursing, and the desire to participate in the study. To determine the required sample volume at confidence level of 95% with the assumption of r = 0.2 [41], the sample volume of 260 nurses was estimated using the related formula. Considering participants attrition rate of 10%, 287 nurses were selected for the study. Eleven participants did not return the instruments, and 26 deficiently filled instruments were excluded from statistical analysis so that, ultimately, 250 participants entered the study.

### Data collection tools

To cull the data, in addition to the Demographic Questionnaire, Ethical Leadership Questionnaire (ELQ) [47], Hospital Ethical Climate Survey (HECS) [48], and Organizational Citizenship Behavior Scale [8], were also used.

## Demographic questionnaire

The Demographic Questionnaire included personal information such as age, gender, work experience, and type of employment.

## Ethical leadership questionnaire (ELQ)

To measure the rate of ethical leadership, the ELQ of Emadifar, (2010) was used. This 41-item tool consisted of 5 categories including “honesty and integrity”, “setting activities in an ethical framework”, “trust”, “efforts to enhance the employees”, and “expressing dissenting opinions” using a 4-point Likert scale. Ranging from “I completely agree=4 to “I completely disagree = 1. The total score of this inventory ranged between 41 and 164 where a greater score on the instrument indicated higher quality of ethical leadership in the organization. A nursing manger obtaining a score higher than the average score (102.5) is recognized as an individual whose managerial behavior corresponds to the ethical leader’s behavioral characteristics. The internal consistency reliability of this instrument was confirmed by estimated α = 0.98 using Cronbach’s α correlation coefficient [47, 49]. In this study, Cronbach’s α coefficient was 0.84 for EL Questionnaire.

## Hospital ethical climate survey (HECS)

HECS was developed by Olson to assess nurses’ perceptions of the ethical climate of their workplace. HECS is a 26-item self-report instrument with 5 subscales including relationships with peers, patients, managers, hospital, and physicians. A 5-point Likert scale was used to assess the responses. Each item in the tool ranged from “almost never = 1″ to “almost always = 5″. In this way, the minimum score on the tool for each individual was 26 while the maximum score was 130. In Olson’s study, the content validity of this instrument was obtained as 0.89 and the internal consistency correlation coefficient was estimated as 0.91 for the whole instrument using Cronbach’s α correlation coefficient [48]. The Cronbach’s α for the Ethical Climate Questionnaire was obtained as 0.89 in the present study.

## Organizational citizenship behavior scale

This 20-item scale was developed by Podosakoff et al. (1990) and consisted of 5 categories including “altruism”, “conscientiousness”, “sportsmanship”, “courtesy” and “civic virtue”, and “social manners”. The scores on this instrument ranged between 1 and 5 where a higher score suggested a higher and better level of OCB [8]. The Cronbach’s α correlation coefficient of this instrument was obtained as 0.79 in the present study which was approved for the study.

### Data analysis

The gleaned data were analyzed with SPSS18 and AMOS software version 24 Descriptive statistical methods (mean and standard deviation) were used to describe the data. Spearman’s correlation coefficient was used to determine the relationship between variables. Regression analysis and the structural equation model were used to investigate the simultaneous relationship of EL, EC and OCB.

## Results

Our findings demonstrated that the mean age of the study participants was 32.26 ± 7.1 years with a work experience of 7.7 ± 6.9 years. In addition, 82.2% of the participants (207 nurses) were female.

From nurses’ perspective, the total mean score of EL style was 133.74 ± 21.64, indicating that nursing managers’ EL style was at an acceptable level. From nurses’ perspective, the total mean score of EC was 76.97 ± 19.27 and the mean score of OCB was 80.75 ± 16.22 namely at a high level (Table 1).

**Table 1:** Mean scores of variables’ from the nurses’ perspective

Variables	Mean	SD
Ethical leadership	133.74	21.64
Ethical climate	76.97	19.27
OCB	80.75	16.22

Spearman’s correlation coefficient revealed a significant correlation between “EL” and “EC” (*P* < 0.001, r = 0.65). The findings of the study further showed that there was a slightly significant correlation between “EL” and “OCB” (*P* = 0.04, r = 0.09). There was also a significant correlation between “EC and” and “OCB” (P < 0.001, r = 0.61) (Table 2).

**Table 2:** Matrix of correlation coefficients of research variables (Spearman correlation test)

Variables		Ethical Leadership	Ethical climate	Organizational citizenship behavior
Ethical Leadership	Correlation Coefficient	1	0.65	0.092
	*P*-Value		< 0.001	0.046
	Number		250	250
Ethical climate	Correlation Coefficient	0.65	1	0.61
	P-Value	< 0.001		< 0.001
	Number	250		250
Organizational citizenship behavior	Correlation Coefficient	0.092	0.61	1
	P-Value	0.046	< 0.001	
	Number	250	250	

*P*-Value < 0.05

To explain the OCB as the dependent variable in the relationship between EL and EC, an estimation of the regression model was used. The results of regression analysis showed that the multiple correlation coefficients between EL and the EC of nurses, with the OCB in the whole sample obtained 0.84. In addition, the coefficient of determination (squared multiple correlation coefficient) of the predictive variables is about 70% (Table 3).

**Table 3:** Summery of regression model

Model Summary^b^				
Model	R	R^2^	Adjasted R^2^	SE of the estimate
1	0.842 ^a^	0.709	0.707	8.77

^a^Predictors: (Constant), Ethical Leadership, Ethical Climate.

^b^Dependent Variable: Organizational Citizenship Behavior

**Fig. 2:**
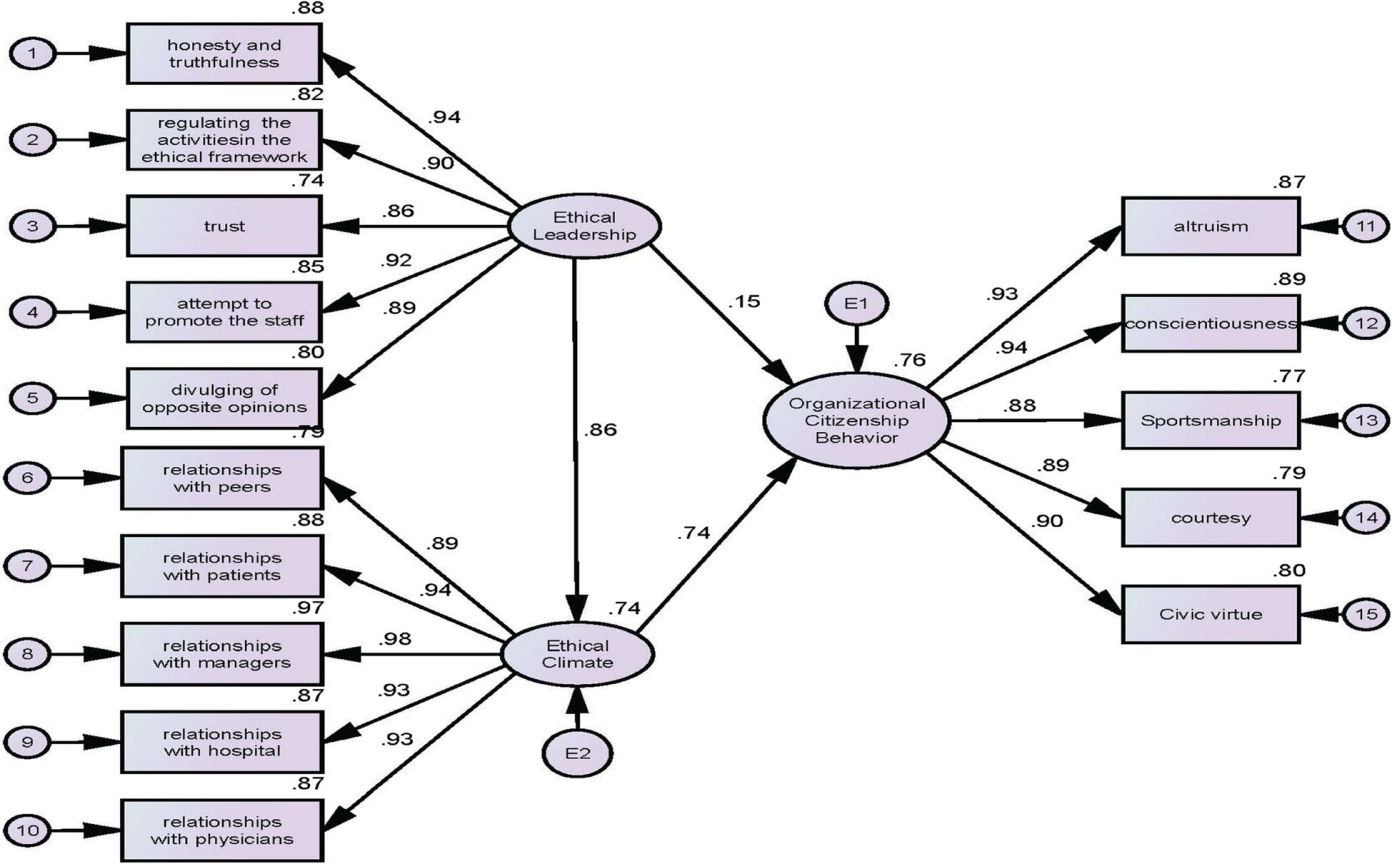
model

Finally, with regard to the results using AMOS software (structural equation modelling), that overall indicators showed the model fit by the data, thus we can conclude the collected data well support the model. The results showed that EL had a positive effect on the EC of nurses and OCB (Table 4, Fig. 2).

**Table 4:** Model fitness

Model	χ^2^_(df)_	CFI	RMSAE	RMSAE
1	530.656 (87) p < 0.001	1	0.14	0.14

## Discussion

The aim of this study was to investigate the relationship between nursing managers’ EL from nurses’ perspective and EC with nurses’ OCB.

The results of correlation between EL, EC and nurses’ OCB showed a significant positive correlation (*p* < .001). The results of regression analysis showed that the multiple correlation coefficients between the EL and EC of nurses, with OCB are obtained (r = 0.842). In addition, the coefficient of determination (squared multiple correlation coefficient) of the predictive variables is about 70%. This means that 70% of the variance of the dependent variable (OCB) can be predicted by the independent variables (in this study: EL from the perspectives of nurses and the EC of nurses). There is no similar study in the literature to our knowledge, and the study can be considered an innovation.

According to our findings, there was a significant correlation between “EL”, and “EC” (*P* < 0.001). These results were also in line with other studies [17, 37, 50, 51]. Although most previous studies have been conducted in various settings such as universities, companies, firms, schools, and in industry, the present study was carried out with its specific conditions in a different milieu, i.e., the field of health and treatment in hospitals with its specific clients, namely the patients. Hence, the variables of EC are of more importance in health-care organizations.

Undoubtedly, an ethically oriented approach in leadership bestows many benefits on organizations, the importance of which is manifested in the health care management system and in the relationships between colleagues. In fact, similarity of duties, work environment, and specific hospital conditions necessitate the existence of a close friendly atmosphere among nurses, staff, physicians, and patients; because of this proximity, many conflicts and ethical issues are prone to occur. It appears that when an EC governs the hospital, and managers are committed to ethical principles, the nurses are expected to show ethical behaviors. This could result in creating a positive social climate, teamwork, and resolving problems in the workplace [52].

According to the findings of this study, a significant correlation exists between EC and OCB (*p* < .001), so that the more favorable the atmosphere governing the organization, the greater the manifestation of OCB in that organization. The study results were in line with Kolade et al. and Huang et al. [11, 37].

Today, the topic of organizational EC and OCB is rendered as an important issue in all organizations. Consequently, the kind affectionate individuals are not only logically liked by their workmates, but also are looked at as reliable trusted individuals. In this way, kindness, affection, and humanity increase the strong relations between citizens.

Furthermore, the findings of this study demonstrated a significant correlation between EL and OCB (*p* < .001). In line with this research, the study by Baharloo et al. on analyzing the correlation between EL and OCB showed a significant correlation between these variables, which is consistent with our findings [41]. The reason for the effect of EL on the formation of citizenship behavior may be attributed to the point that a positive just behavior towards the staff drives them to ponder on their relations with citizens and preserve the leaders’ services as the maintenance of attitudes required for implementing organizational goals. However, in contrast with our results, Sabzipoor et al. showed that the improvement of organizational climate has no effect on OCB [53]. The reason for this disparity may be the difference in the research community.

Our findings indicated that nursing managers’ EL is at a high level from nursing perspective which was consistent with the results of other researchers [49, 54–57], although it went against the results of a research by Avatefi Monfared et al. [58] and Abbas Pour et al. [59]. The reason for this disparity may be the difference in studied units, different research tools, and cultural and value differences. Many theories of leadership emphasize that any organization requires leaders that not theoretically but practically design an optimistic landscape in the organization through observing ethical values, respecting the staff, and showing affection for them [60].

The results showed that the EC level is at a desirable level. In this regard, the results of many studies revealed that the nurses’ overall perception of EC of the organization was positive [39, 61]. An inappropriate EC can definitely affect the staff’s behavior and expose the organization to crisis. Based on these results, it is recommended that nurse managers develop EC in their hospitals. They should further foster EC by role modeling in conversations with nurses.

The results showed that the level of OCB is high in the hospitals where the research was carried out. These findings are consistent with the results of the study by Mayel Afshar et al. [62]. It appears that allowing the contribution of the personnel in the organizational affairs and exchange of opinions among the managers and the staff would lead to the creation of a friendly environment along with confidence, reciprocal reliance, and increased rate of organizational civil behavior resulting in fostered organizational efficiency and output.

This study had a few limitations. First, personal feelings and emotions of participants will undoubtedly influence their responses to provide real responses to the questions which have a socially desirable answer. Another limitation was the inability to control the mediating variables such as factors affecting the accuracy and concentration of staff when delivering the questionnaires because of the large amount of work.

Our findings may not be generalized to nonteaching, referral, and private hospitals. The authors suggest that such research be done in referral and private hospitals, and with other healthcare professionals in Iran.

## Conclusion

There was a significant correlation between “EL” and “EC” and also between EC and OCB. The findings also demonstrated a significant correlation between EL and OCB. In addition, 70% of the variance of the dependent variable (OCB) can be predicted by EL and the EC from the perspectives of nurses. Therefore, some suitable conditions may be provided to achieve organizational efficacy.

The study of this research provides direction for targeting interventions to improve nurse manager’s ethical leadership for developing staff’s organizational citizenship behavior and ethical climate. Providing training and promotion of such a leadership style in the form of workshops and periodic counseling can be effective. This should also be considered in the nursing education program. This paper contributes to redressing the lack of educational opportunities, and consequently, enhancing nurses’ managerial competencies.

## Data Availability

The datasets generated and/or analyzed during the current study are not publicly available due to an agreement with the participants on the confidentiality of the data but are available from the corresponding author on reasonable request.

## Abbreviations

EC: Ethical Climate
EL: Ethical Leadership
ELQ: Ethical Leadership Questionnaire
HECS: Hospital Ethical Climate Survey
OCB: Organizational Citizenship Behavior
SBMU: Shahid Beheshti University of Medical Sciences, Tehran, Iran
SCT: Social cognitive theory
